# Sodium alendronate loaded poly(l-lactide- *co*-glycolide) microparticles immobilized on ceramic scaffolds for local treatment of bone defects

**DOI:** 10.1093/rb/rbaa012

**Published:** 2020-03-30

**Authors:** Łucja Rumian, Cornelia Wolf-Brandstetter, Sina Rößler, Katarzyna Reczyńska, Hanna Tiainen, Håvard J Haugen, Dieter Scharnweber, Elżbieta Pamuła

**Affiliations:** r1 Faculty of Materials Science and Ceramics, Department of Biomaterials and Composites, AGH University of Science and Technology, Al. A. Mickiewicza 30, Krakow 30-059, Poland; r2 Technische Universität Dresden, Institute of Materials Science, Max Bergmann Center of Biomaterials, Budapester Str. 27, Dresden 01-069, Germany; r3 Department of Biomaterials, Institute for Clinical Dentistry, University of Oslo, Geitmyrsveien 71, Blindern, P.O. Box 1109, Oslo NO-0317, Norway

**Keywords:** ceramic scaffolds, sodium alendronate, osteoblasts, osteoclastogenesis, collagen, critical-size defect, poly(l-lactide-co-glycolide), microparticles

## Abstract

Bone tissue regeneration in critical-size defects is possible after implantation of a 3D scaffold and can be additionally enhanced once the scaffold is enriched with drugs or other factors supporting bone remodelling and healing. Sodium alendronate (Aln), a widely used anti-osteoporosis drug, exhibits strong inhibitory effect on bone resorption performed by osteoclasts. Thus, we propose a new approach for the treatment of bone defects in craniofacial region combining biocompatible titanium dioxide scaffolds and poly(l-lactide-*co*-glycolide) microparticles (MPs) loaded with Aln. The MPs were effectively attached to the surface of the scaffolds’ pore walls by human recombinant collagen. Drug release from the scaffolds was characterized by initial burst (24 ± 6% of the drug released within first 24 h) followed by a sustained release phase (on average 5 µg of Aln released per day from Day 3 to Day 18). *In vitro* tests evidenced that Aln at concentrations of 5 and 2.5 µg/ml was not cytotoxic for MG-63 osteoblast-like cells (viability between 81 ± 6% and 98 ± 3% of control), but it prevented RANKL-induced formation of osteoclast-like cells from macrophages derived from peripheral blood mononuclear cells, as shown by reduced fusion capability and decreased tartrate-resistant acid phosphatase 5b activity (56 ± 5% reduction in comparison to control after 8 days of culture). Results show that it is feasible to design the scaffolds providing required doses of Aln inhibiting osteoclastogenesis, reducing osteoclast activity, but not affecting osteoblast functions, which may be beneficial in the treatment of critical-size bone tissue defects.

## Introduction

Although bone tissue has the intrinsic capacity for regeneration, extensive bone defects originating from illnesses, tumour resection, trauma or bacterial infections do not heal without intervention [1]. Worldwide, the market for bone grafts is estimated to exceed $3 billion and reconstruction of craniofacial injuries accounts for 13% of the market (over $390 million) [2]. Facial bone defects not only pose a serious health problem for the patient, but also render numerous physiological and sociological difficulties, including lack of social acceptance or speech impediment [2, 3]. Therefore, in the most severe cases of craniofacial bone damage, a supportive structure (scaffold) combined with appropriate pharmacotherapy is necessary to promote bone regeneration and healing [4].

The use of autologous bone graft is still the gold standard in bone tissue repair. However, as the use of autografts can increase the risk of an additional injury, scarring and morbidity, the autographs are believed to be soon overtaken by allografts, xenografts or synthetic bone replacement materials [2, 5–7]. Numerous biomaterials including natural and synthetic polymers (e.g. collagen, chitosan, polylactide, poly(lactide-*co-*glycolide) (PLGA)), metals (e.g. gold, titanium) or ceramic materials have been used for the regeneration of craniofacial defects [3]. Ceramic materials (e.g. hydroxyapatite, tricalcium phosphate or bioactive glasses) gain particular interest in this application due to their chemical and structural similarity to the native bone [3, 8–10]. However, their poor mechanical properties (compressive strength lower than 2 MPa [11]) compared with those of bone have hindered their clinical applications; as a result they are practically used in the non-load-bearing sites [12, 13]. On the other hand, titanium dioxide (TiO_2_) scaffolds have been shown to be a viable option for bone tissue defect treatment [14–20]. It was already evidenced that the TiO_2_ scaffolds with porosity over 90% and compressive strength of 3.4 MPa (similar to the strength of healthy trabecular bone, i.e. 2–12 MPa) were biocompatible and enhanced tissue regeneration *in vivo* [14, 17–20]. Such highly porous TiO_2_ scaffolds implanted in extraction sockets of minipigs jaws provided adequate support for bone regeneration and after 6 weeks of healing newly formed bone tissue was found in the whole volume of the implanted scaffolds [15].

With the recent developments in medicine and the understanding of cellular and molecular basics of bone regeneration processes, it is possible to enrich bone replacement materials with drugs or other biologically active moieties to support bone growth. Bone morphogenetic proteins (BMPs) are known to induce the proliferation and osteogenic differentiation of mesenchymal stem cells, but as numerous studies have already reported, due to adverse effects their clinical application is still limited [21–24]. On the other hand, bone regeneration and remodelling can be enhanced once bone resorption is inhibited [25].

Bisphosphonates (BPs) with their high affinity for bone calcium phosphates and long safety record, constitute the largest class among drugs preventing bone resorption [26, 27]. The nitrogen-containing BPs act by binding to an enzyme in the 3-hydroxy-2-methylglutaryl-CoA reductase pathway (i.e. the mevalonate pathway), thus blocking the prenylation of small GTPases. The latter are essential for osteoclast functionality and survival and thus inhibition of bone resorption by suppressing osteoclast activation and inducing osteoclast apoptosis [26, 28]. BPs are inexpensive and commonly prescribed to treat bone diseases such as osteoporosis, Paget’s disease of bone or osteolytic bone metastases [29–31]. Unfortunately, systemic oral administration of BPs provokes several side effects (e.g. gastrointestinal problems or osteonecrosis of the jaw after long-term use) [31–38].

One of the most widely used BPs is sodium alendronate (Aln) [39]. After absorption in the bone, Aln has an estimated terminal elimination half-life of 10 years [40]. Aln increases bone formation, enhances osteoblasts proliferation, maturation and mineralization and leads to inhibition of osteoblast apoptosis [41, 42]. Several clinical trials have already been carried out which showed promising results for the treatment of periodontal disease with Aln [39]. A local administration of Aln seems to be a good alternative to minimize the side effects of conventional systemic drug administration in combination with strong local effects.

Therefore, there is a need to develop a local delivery system for Aln, which will allow for sustained and controlled release of the drug. A resorbable aliphatic polyester PLGA was chosen as encapsulating material for Aln. It is a copolymer approved by European Medicines Agency and Food and Drug Administration, and its hydrolytic degradation rate can be adjusted (e.g. by chain structure, molecular weight, lactide to glycolide molar ratio, crystallinity); PLGA microcarriers can be easily manufactured using emulsification as well [43, 44]. It was already demonstrated that immobilization of PLGA microparticles (MPs) loaded with either vancomycin or gentamicin on highly porous TiO_2_ scaffolds provided them with antibacterial properties (effective inhibition of *Staphylococcus* spp. proliferation), while the system was cytocompatible with osteoblast-like cells (MG-63) [14, 17].

The purpose of this study was to improve the bioactivity of highly porous TiO_2_ scaffolds by providing therapeutic function, so that the scaffold does not only assures mechanical support for the cells, but also delivers drugs enhancing bone tissue regeneration. Thus, Aln-loaded PLGA MPs were immobilized on the pore walls of TiO_2_ scaffolds in order to develop a system supporting osteoblast adhesion and proliferation and suppressing osteoclast activity. Human recombinant collagen was used to bind MPs to the surfaces of the scaffolds pore walls. To confirm utility of the scaffolds drug release kinetics and cytocompatibility in contact with osteoblast-like cells were tested. Furthermore, the influence of the scaffolds on model bone resorbing cells was studied, to confirm inhibitory effect of released Aln on osteoclast-like cells. Modified scaffolds can be potentially used for regeneration of critical-size defects in craniofacial region.

## Materials and methods

### Materials

TiO_2_ powder (Kronos 1171, Kronos Titan GmbH, Leverkusen, Germany), polyurethane foams (60 ppi, Bulbren S Eurofoam GmbH, Wiesbaden, Germany), sodium hydroxide (NaOH, 1 M solution) and hydrochloric acid (HCl 0.1 M and 1 M solutions; Sigma-Aldrich, Norway) were used to fabricate TiO_2_ scaffolds. The MPs were made from PLGA (molar ratio of L-lactide to glycolide comonomers 85:15, Mn = 100 kDa, d = 2.1; synthesized at the Center of Polymer and Carbon Materials, Polish Academy of Sciences, Zabrze, Poland) [45]. Aln was a gift from Polpharma S.A., Poland (batch no.: 504041227). Polyvinyl alcohol (PVA, Mowiol 4-88, Sigma-Aldrich, Poland), human recombinant collagen (Bornstein and Traub Type I, recombinant, expressed in *Nicotiana tabacum*), orthophthaldialdehyde (OPA), dichlomethane (DCM) were from Sigma-Aldrich, Poland. Methanol, 2-mercaptoethanol and isopropanol (analytical grade) were purchased from Sigma-Aldrich, Norway. Ultra-high quality water (UHQ-water) was produced using a Purelab system, Elga, UK shortly before use.

### Scaffolds and MPs preparation and modification

TiO_2_ scaffolds were obtained by polymer sponge replication technique and sintering as shown in our previous studies [14, 17]. In short, thermal treatment parameters were as follows: (i) heating up to 450°C at a rate of 0.5°C/min and keeping at 450°C for 1 h; (ii) heating at a rate of 0.5°C/min from 450°C to 1100°C and keeping at 1100°C for 5 h; and (iii) heating at a rate of 3°C/min from 1100°C to 1500°C and sintering at 1500°C for 20 h. As a result cylindrical scaffolds 12 mm both in height and diameter were obtained.

Aln-loaded MPs were manufactured by solid/oil/water method [14, 17]. In brief, 5 mg of Aln was emulsified in 3 ml of 1.67% (w/v) PLGA solution in DCM (theoretical Aln loading: 10% (w/w)). Afterwards, the oil-in-water emulsion was prepared by mechanical stirring of the primary emulsion and 15 ml of 4% PVA aqueous solution at 1000 rpm. Thereafter the emulsion was stirred at 500 rpm overnight to get rid of DCM remnants. After centrifuging (2400 × g, 10 min, 4°C) the MPs were resuspended in UHQ-water three times to remove PVA, then lyophilized (24 h, 0.03 Torr, −50°C, Labconco Freezone). The MPs were stored at 4°C.

Defined amount of MPs (2.6 mg) suspended in 400 μl of collagen aqueous solution (40 μg/ml) was introduced on each TiO_2_ scaffold. The amount MPs was adjusted taking into account drug loading efficiency value (calculated according to the Equation (2), see below) in such a way that amount of Aln per scaffold was 200 μg.

### Sample characterization

The morphology of MPs was evaluated under Axiovert 40 optical microscope (Carl Zeiss, Germany) as well as under scanning electron microscope (SEM, Hitachi TM3030, Hitachi High Technologies Corp., Tokyo, Japan with acceleration voltage 10 kV). The diameter of MPs (*n* = 300) was measured using a software provided by the microscope manufacturer AxioVs40 v. 4.7.1.0 and with the ImageJ software (NIH, USA). To measure the amount of encapsulated drug within the MPs, supernatant from MPs centrifugation was collected. Aln concentration was determined using an OPA test [14, 46] with fluorescence reading (*λ*_ex_ − 340 nm, *λ*_em_ − 455 nm, Lambda 25 UV/Vis, Perkin Elmer, USA). Encapsulation efficiency and loading efficiency were calculated according to Eqs. (1) and (2), respectively:

(1) $$\%\;\mathrm{Encapsulation}\;\mathrm{efficiency}=\;\frac{\mathrm{Mass}\;\mathrm{of}\;\mathrm{drug}\;\mathrm{in}\;\mathrm{MPs}}{\mathrm{Initial}\;\mathrm{mass}\;\mathrm{of}\;\mathrm{drug}\;\mathrm{in}\;\mathrm{the}\;\mathrm{system}}\cdot 100\%$$

(2) $$\%\;\mathrm{Loading}\;\mathrm{efficiency}=\;\frac{\mathrm{Mass}\;\mathrm{of}\;\mathrm{drug}\;\mathrm{in}\;\mathrm{MPs}}{\mathrm{Mass}\;\mathrm{of}\;\mathrm{MPs}}\cdot 100\%.$$

All experiments were performed in triplicate and the results were expressed as mean ± standard error of the mean (SEM).

Microstructure of the scaffolds (as received and after 24 h and 72 h incubation in phosphate buffered saline – PBS) was observed using SEM (Nova Nano SEM 200, FEI Company Europe, acceleration voltage 18 kV). Prior to examination the samples were sputter-coated with carbon layer to make them conductive.

### *In vitro* Aln release study

*In vitro* Aln release study was done in PBS (pH = 7.2) using semipermeable dialysis membranes (Zellu TransRoth, MWCO 12 kDa). One scaffold (average scaffold mass was 61 ± 6 mg) was put in a dialysis bag with 1 ml PBS, sealed and placed in the vial containing 3 ml PBS and shaken (50 rpm) at 37°C. At fixed time intervals (1 h, 8 h, and 1, 2, 3, 7, 11, 16, 22, 44, 50 and 62 days), 1 ml of sample aliquots were collected and 1 ml fresh PBS was added [9]. Aln concentration was measured with OPA method as described above.

### *In vitro* tests with osteoblast-like MG-63 cells

The scaffolds were sterilized using hydrogen peroxide plasma (Sterrad 120, ASP, J&J, USA). Cytocompatibility was assessed by testing the viability of MG-63 cells (European Collection of Cell Cultures, Salisbury, UK) cultured in the extract from samples incubated in Eagle’s minimal essential medium (EMEM, PAN BIOTECH, Germany) supplemented with 10% foetal bovine serum (FBS), 100 U/ml penicillin, 100 µg/ml streptomycin and 0.1% sodium pyruvate (PAA, Austria) [9]. The extract was obtained by the scaffolds incubation in EMEM for 24 h at 37°C (one scaffold was incubated in 2 ml medium; average scaffold mass was 61 ± 6 mg). Extracts from the reference scaffolds containing empty MPs (Sc) and MPs loaded with Aln (Sc Aln) were prepared. The extracts were diluted in EMEM by factors of 1:1 (undiluted), 1:2, 1:4 and 1:8. Additionally, different concentrations of Aln dissolved in EMEM were prepared: 100, 50, 10, 5 and 1 µg/ml. Cells cultured in medium (EMEM) acted as control. MG-63 cells were cultured at initial density 5 × 10^5^ cells in a 48-well plate (Nunclon, Germany) for 24 h and then medium was replaced by the undiluted or diluted extracts or Aln solutions (1 ml). AlamarBlue reagent (resazurin based, Sigma-Aldrich, Poland) was used to determine cell metabolic activity 1, 3 and 6 days post-treatment. Reduction of AlamarBlue was measured based on fluorescence intensity (*λ*_ex_—530 nm, *λ*_em_—590 nm, FLUOstar Omega, BMG Labtech). Cell attachment, distribution and viability were also evaluated after live/dead staining with calcein AM/propidium iodide (Sigma-Aldrich, Poland) under Axiovert 40 inverted fluorescence microscope (Carl Zeiss, Germany) [14]. Code names of extracts and drug solutions can be found in Table 1.

**Table 1:** Descriptions of samples–extracts from scaffolds and drug solutions for *in vitro* tests on MG-63 cells and model osteoclasts derived from human peripheral blood mononuclear cells (PBMC) cultured in EMEM and αMEM, respectively

Drug	Scaffolds/ drug (S/D)	Drug on scaffold (µg)	Dilution	Drug concentration (µg/ml)	Code
None	Scaffold		1:1		Sc 1:1
			1:2		Sc1:2
			1:4		Sc 1:4
			1:8		Sc 1:8
Aln	Scaffold	200	1:1		Sc Aln 1:1
			1:2		Sc Aln 1:2
			1:4		Sc Aln 1:4
			1:8		Sc Aln 1:8
Aln Drug				100	Aln 100
				50	Aln 50
				10	Aln 10
				5	Aln 5

				2.5	Aln 2.5
				1	Aln 1
				0.5	Aln 0.5
Medium					EMEM
					αMEM

### *In vitro* tests with human monocyte-derived osteoclast-like cells

For cell isolation Leucosep^TM^ tubes (Greiner Bio-One) filled with 15.5 ml of Ficoll-PaqueTM Plus (GE Healthcare; density 1.077 ± 0.001 g/ml at 20°C) were used. Human peripheral blood mononuclear cells (PBMC) were isolated from the buffy coats of healthy adult donors (purchased from the blood donation service in Dresden, Germany)—the pooled cells from two different donors were used in the experiment. For blood dilution and isolation, PBS containing 2 mM ethylenediaminetetraacetate (EDTA) and 0.5% bovine serum albumin (both Sigma-Aldrich) (PBS E/B) was prepared. Monocyte adhesion medium consisted of minimum essential medium, alpha modification (αMEM) containing 7.5% heat-inactivated FBS, 7.5% human serum AB (CCpro, off-the-clot), 100 U/ml penicillin (Biochrom), 100 µg/ml streptomycin and 2 mM L-glutamine (Biochrom). For cell differentiation macrophage-colony stimulating factor from recombinant mouse (M-CSF) (Sigma-Aldrich) and recombinant human receptor activator of nuclear factor-kappa B ligand (RANKL) (R&D Systems) were used, both at 25 ng/ml concentration.

The extract was obtained by the scaffolds incubation in αMEM for 24 h at 37°C (one scaffold was incubated in 2 ml medium; average scaffold mass was 61 ± 6 mg). The extract was diluted in αMEM by factors of 1:1 (undiluted), 1:2, 1:4 and 1:8. Additionally, different concentrations of Aln (5, 2.5, 1 and 0.5 µg/ml) dissolved in medium were prepared. The sample code names were the same as those for the experiment with MG-63 cells except for control αMEM medium (Table 1).

The buffy coats were diluted at ratio 1–1 (v/v) in PBS E/B, then they were transferred into prefilled Leukosep^TM^ tubes and centrifuged at 750 g for 15 min without break. The upper yellowish phase containing plasma and the platelets was aspirated, while the white leukocyte band was harvested and resuspended in ice-cold PBS E/B and centrifuged at 250 × g and 4°C for 15 min; washing step was repeated once more. After last washing step, the pellet was resuspended in ice-cold sterile water for 30 s to remove erythrocytes followed by immediate addition of 48 ml of ice-cold PBS E/B. After repeated centrifugation, the cells were resuspended in 0.5 ml αMEM and seeded into 48-well plates at a concentration of 2 × 10^5^ cells per well. The monocytes obtained from PBMC by adherence to tissue culture polystyrene (TCPS) well plates were incubated for 24 h in αMEM. Then the medium was aspired and replaced by the extracts or drug solutions; growth factors (MCSF and RANKL) were added. Medium containing required extracts or drug solutions was exchanged twice a week. The experiments were performed in triplicate. After 3, 8 and 15 days of culture the plates were washed with PBS and stored at −80°C until further analysis of DNA content and tartrate-resistant acid phosphatase 5b (TRAP5b) activity. After 15 days, samples for microscopic evaluation were fixed and stained with Leukocyte Acid Phosphatase (TRAP) Kit (Sigma-Aldrich) according to the manufacturer protocol. To estimate the degree of cell fusion, total number of nuclei and nuclei within multinucleated giant cells (≥3 nuclei/cell) were counted. At least three images were analysed for each type of the samples. The fusion index was calculated using the formula: fusion index (%) = total number of nuclei within giant cells/total number of nuclei counted × 100 [47, 48].

Frozen cell culture plates were thawed for 20 min on ice followed by the addition of 300 µl of 1% Triton X-100 in PBS. The plates were shaken for 50 min, then cell lysates were sonicated in ice-cooled ultrasonic cleaner (VWR, USA) for 10 min.

Cell lysates (10 µl) were evaluated by using the Quant-iT™ PicoGreen dsDNA Reagent (Invitrogen, Darmstadt, Germany) for DNA quantification (190 µl); the reagent was diluted 1:800 in TE buffer (10 mM TRIS and 1 mM EDTA; pH 7.5) and incubated for 7.5 min in the dark. The intensity of fluorescence was measured (*λ*_ex_—485 nm, *λ*_em_—535 nm, Tecan SpectraFluor Plus microplate reader, Crailsheim, Germany).

Osteoclast differentiation was evaluated by the measurement of tartrate-resistant acid phosphatase (TRAP) 5b activity using naphthol-ASBI phosphate (N-ASBI-P, Sigma-Aldrich) as a substrate according to a modified protocol of Janckila et al. [49]. Fifty microlitre of TRAP 5b reaction buffer consisting of 2.5 mM N-ASBI-P in buffer containing 100 mM sodium acetate (Merck), 50 mM sodium tartrate (Fluka), 2% NP-40 (Sigma) and 1% ethylene glycol monomethyl ether (Sigma) adjusted to pH 6.1, was added to cell lysates (10 µl), followed by incubation at 37°C for 30 min. The reaction was stopped by 125 µl 0.1 M NaOH (Sigma) solution and the intensity of fluorescence was measured (*λ*_ex_—405 nm, *λ*_em_—535 nm, Tecan SpectraFluor Plus microplate reader, Crailsheim, Germany). Different TRAP concentrations (BoneTRAP) were used to obtain calibration curve. TRAP 5b activity was related to DNA content of the respective samples.

### Statistical analysis

A one-way analysis of variance test was performed for comparison of different data groups, followed by Tukey’s *post hoc* test. Normality assumption and equal variance were verified by setting the *P*-values to 0.05, 0.01 or 0.001 using the Shapiro–Wilk’s and Levene Median tests, respectively (OriginPro 2015 Sr2, OriginLab Corp., USA).

## Results

### Physico-chemical and microstructural properties of MPs and scaffolds

**Figure 1:**
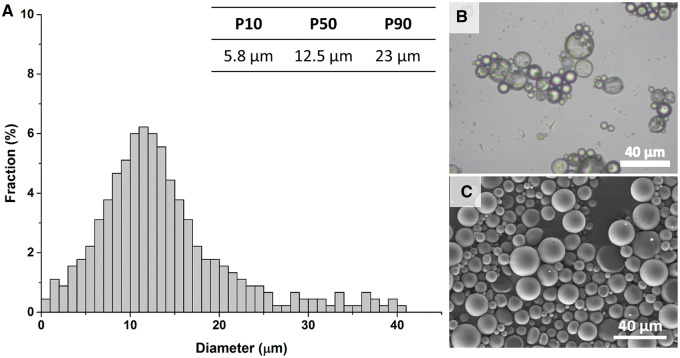
Size distribution of Aln-loaded PLGA MPs (**A**), morphology of MPs under optical microscope (**B**) and under SEM (**C**). Scale bar = 40 μm (B, C).

Aln-loaded MPs obtained by solid/oil/water emulsification solvent evaporation technique were smooth and spherical (Fig. 1B and C). The histogram presenting the diameter of MPs showed that the size of 80% of MPs was within the range of 5.8–23 µm, with the median of 12.5 µm (Fig. 1A). The encapsulation efficiency for Aln-loaded MPs was 71 ± 6% and the drug loading efficiency was 7.7 ± 0.8%.

**Figure 2:**
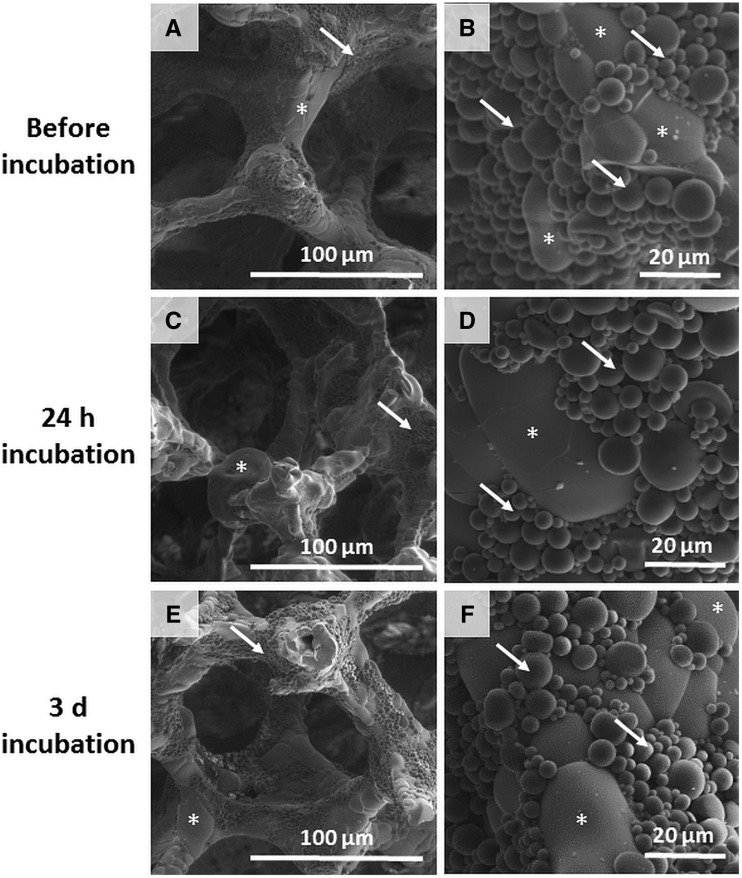
SEM images of TiO_2_ scaffolds decorated with MPs loaded with Aln (200 µg) at two magnifications before (**A, B**), after 24 h (**C, D**) and after 3 days (**E, F**) incubation in PBS; stars show scaffold, arrows show MPs. Scale bars = 100 μm (A, C, E) and 20 μm (C, D, F).

Aln-loaded MPs were immobilized on the scaffolds surface with the use of collagen solution (40 µg/ml) in such a way that calculated amount of Aln per scaffold was 200 µg. MPs were present on the edges of pore walls as well as on the surface of the struts (Fig. 2A and B). Incubation in PBS for 24 h (Fig. 2C and D) and 3 days (Fig. 2E and F) did not cause significant detachment of the MPs from the scaffolds.

**Figure 3:**
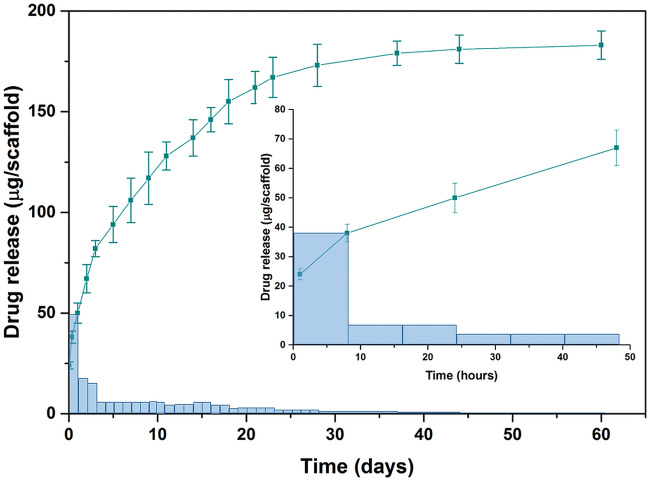
*In vitro* Aln release profiles from Aln-loaded MPs scaffolds: cumulative (squares, mean ± SEM) and increment (bars, dose released per unit time). Insert presents drug release up to 48 h. Bars show released Aln dose per unit time: 1 day for release time from 0 to 62 day and 8 h for release time from 0 to 48 h (insert).

Aln release from the scaffolds was studied in PBS at pH 7.2 (Fig. 3). The initial burst release after 8 h was 38 ± 2 μg (corresponding to 19 ± 1% of total encapsulated drug). After 24 h 48 ± 3 μg (24 ± 6%) of the drug was released. This first phase was followed by an intermediate zero order release phase from Day 3 up to Day 18 with average 5 μg Aln per day. Afterwards up to Day 44, the dosage was constantly decreasing. The released amount of Aln from the scaffolds during 62 days was above 90% of initially encapsulated drug in MPs.

### *In vitro* tests with osteoblast-like cells

**Figure 4:**
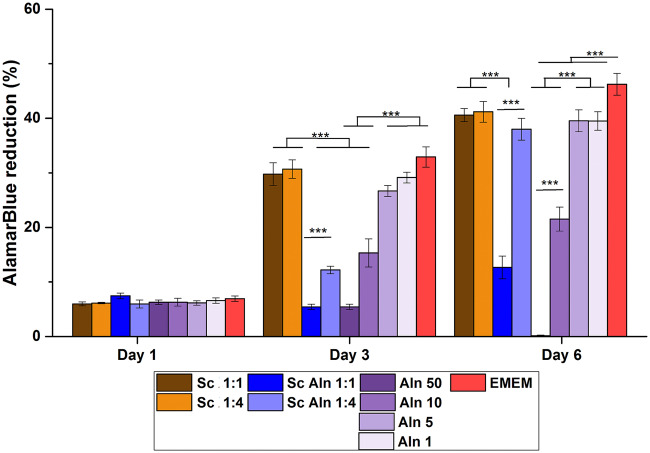
Metabolic activity of MG-63 cells by reduction of AlamarBlue on Days 1, 3 and 6 for extracts diluted in EMEM by the factors 1:1 (undiluted) and 1:4 from reference TiO_2_ scaffolds (Sc, brown columns), TiO_2_ scaffolds containing Aln-loaded MPs attached with collagen (Sc Aln, blue columns). Additionally, results of MG-63 cells cultured in Aln solutions in EMEM are shown (100, 50, 10, 5 and 1 µg/ml Aln, violet columns). as control, activity of cells cultured in EMEM is shown (red columns). Mean ± S.D. ***P < 0.001.

**Figure 5:**
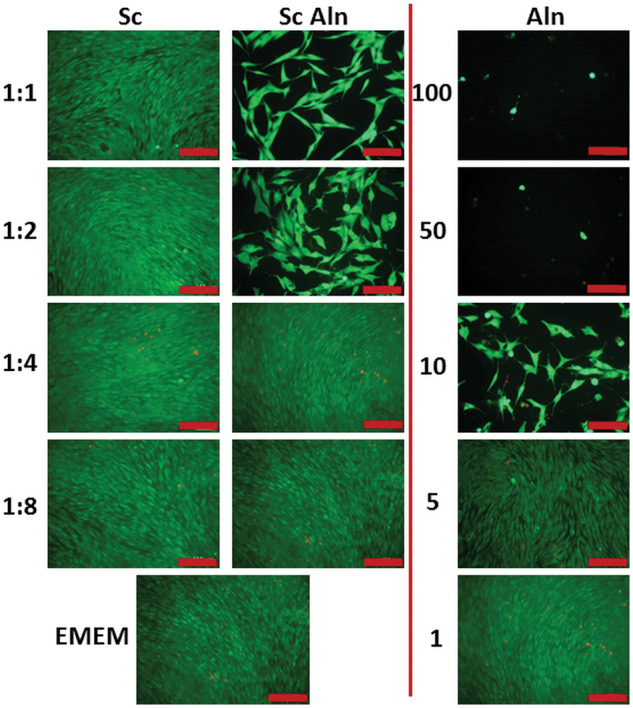
Live-dead staining of MG-63 cells on Day 6 in contact with extracts diluted in EMEM by the factors 1:1 (undiluted), 1:2, 1:4 and 1:8 from TiO_2_ scaffolds (Sc, first column), TiO_2_ scaffolds containing Aln-loaded MPs attached with collagen (Sc Aln, Middle column). Additionally, results of MG-63 cells in Aln solutions in EMEM are shown (100, 50, 10, 5 and 1 µg/ml Aln, last column). as control, activity of cells cultured in pure medium is shown (EMEM, lowest panel on the left). All scale bars = 100 μm.

Figure 4 shows metabolic activity of MG-63 cells assessed using AlamarBlue, whereas Fig. 5 presents live-dead staining of cells cultured in the extracts from studied scaffolds diluted by factors 1:1 (undiluted), 1:2, 1:4 and 1:8. Additionally, also different concentrations of the pure drug dissolved in medium were tested.

Cell viability was the same for all the samples on Day 1 (resazurin reduction in the range of 5.7 ± 0.4–7.6 ± 0.9%, no significant differences between the samples). On Day 3 and Day 6 for reference scaffolds (Sc), i.e. extracts from the scaffolds with empty MPs, cell viability was the same as for the cells cultured in EMEM (29.8 ± 4.2% and 32.2 ± 3.6% for the cells incubated in undiluted extract from the scaffolds and in EMEM, respectively, Fig. 4, brown columns vs. red columns).

Pure drug dissolved in EMEM at the concentration of 50 and 10 µg/ml (Fig. 4, violet columns) decreased viability of the cells after 3 and 6 days both in comparison with cells cultured in extracts from the reference scaffolds as well as in pure medium (EMEM) (about 50% viability for the cells cultured in 10 µg/ml Aln and below 20% for the cells cultured in 50 µg/ml in comparison to untreated cells). It was also observed on live-dead stained samples with addition of 50 and 10 µg/ml Aln (Fig. 5, last column).

On the other hand, on Day 3 cell viability of 5 and 1 µg/ml Aln concentrations was the same as in the extracts from reference scaffolds and for cells cultured in pure EMEM (resazurin reduction of 27.5 ± 1.8% for 5 µg/ml Aln and 28.7 ± 2.1% for 1 µg/ml Aln in comparison to 31.2 ± 3.3% for undiluted extract from reference scaffold and 32.3 ± 3.7% for control cells). On Day 6, there was also no significant difference between cells cultured in 5 and 1 µg/ml Aln concentrations and in the extracts from reference scaffolds (resazurin reduction of 39.2 ± 3.6% for 5 µg/ml Aln and 32.9 ± 3.1% for 1 µg/ml Aln in comparison to 40.1 ± 2.6% for undiluted extract from the reference scaffold).

Live-dead staining on Day 6 shows that cells cultured in pure medium and the extracts from reference scaffolds were alive (less than 2% adhering cells were stained red, i.e. dead) (Fig. 5, Sc, first column and EMEM, bottom). Addition of 100, 50 and 10 µg/ml Aln reduced number of live cells, but addition of 5 and 1 µg/ml Aln did not have an impact on cell culture (Fig. 5, Aln, last column). For scaffolds with Aln-loaded MPs for 1:1 and 1:2 dilutions the number of cells was lower but for higher dilutions, i.e. 1:4 and 1:8 the cells formed a uniform monolayer (Fig. 5, Sc Aln, second column).

### *In vitro* tests with osteoclast-like cells

The aim of the osteoclastogenesis study, starting from monocytes separated from PBMC by adherence to TCPS surface, was to assess if the drug provided with the system is able to prevent formation of the osteoclast-like cells and/or if it can reduce their activity. Based on the results from the experiment with MG-63 showing that cell viability is diminished if Aln concentration is too high (i.e. 100, 50 and 10 µg/ml) tested concentrations of Aln in medium were equal to 5, 2.5, 1 and 0.5 µg/ml.

**Figure 6:**
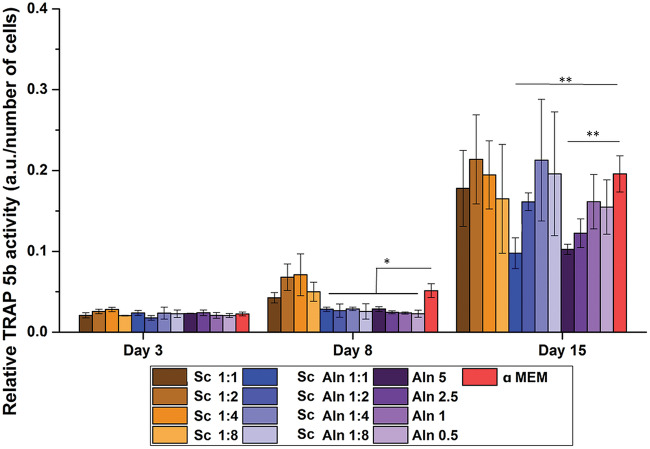
TRAP 5b activity (related to DNA content) on Days 3, 8 and 15 of monocytes derived from PBMCs cultured in extracts diluted in αMEM by the factors 1:1 (undiluted), 1:2, 1:4 and 1:8 from: TiO_2_ reference scaffolds (Sc, brown columns) and TiO_2_ scaffolds containing Aln-loaded MPs attached with collagen (Sc Aln, blue columns). additionally, results for cells cultured in Aln solutions in αMEM are shown (5, 2.5, 1 and 0.5 µg/ml Aln, violet columns). as control, activity of cells cultured in αMEM is shown (red columns). Mean ± S.D. Comparison with αMEM *P < 0.05 and **P < 0.01.

The differentiation of the monocytes into osteoclast-like cells was evaluated by the determination of TRAP 5b activity in culture with different extracts and drug concentrations on Days 3, 8 and 15 (Fig. 6). Since TRAP 5b is expressed by mature osteoclasts but not by monocytes, its activity is an indicator of osteoclastic differentiation. Interestingly, for all Sc Aln extracts and Aln concentrations after 8 days of cell culture the TRAP 5b activity was significantly lower in comparison to control α-MEM (at least 40% reduction for all Sc Aln extracts and 56 ± 5% for Aln alone, *P* < 0.05). After 15 days of culture only cells cultured with 5 µg/ml Aln and in 1:1 extract from the scaffolds with Aln-loaded MPs showed significantly lower TRAP 5b activity as compared to pure medium (50% reduction for undiluted extract from Sc Aln and 46% reduction for Aln 5 µg/ml in comparison to α-MEM, *P* < 0.01).

**Figure 7:**
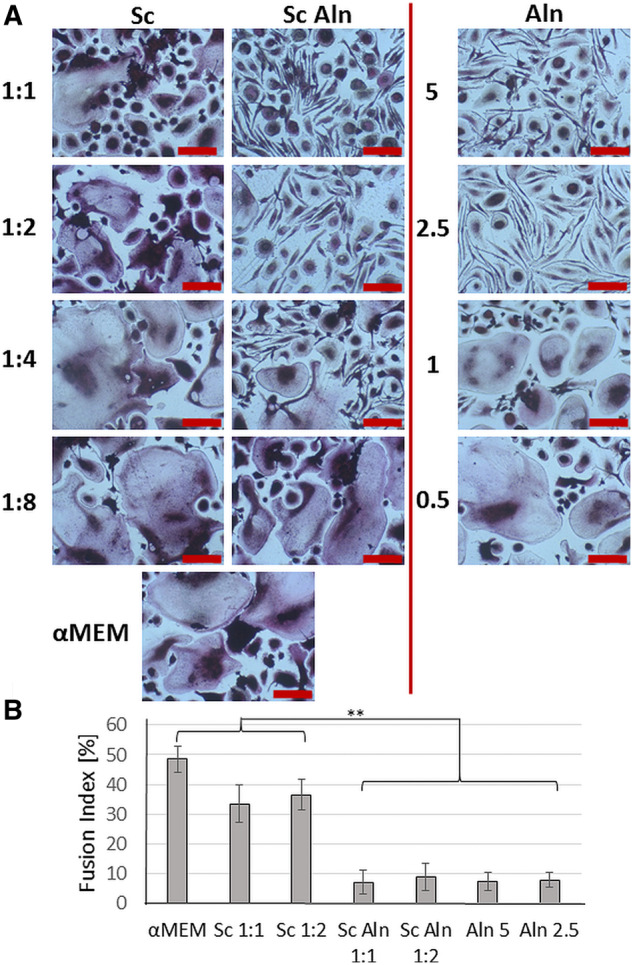
Osteoclast-like cell cultures visualized by TRAP staining (**A**) after 15 days in contact with extracts diluted in α-MEM by the factors 1:1 (undiluted), 1:2, 1:4 and 1:8 from TiO_2_ scaffolds (Sc, first column), TiO_2_ scaffolds decorated with Aln-loaded MPs attached with collagen (Sc Aln, second column). Additionally, results of cells cultured in Aln solutions (5, 2.5, 1 and 0.5 µg/ml Aln, last column) are shown. As a reference, cells cultured in α-MEM medium are presented (lowest panel on the left). All scale bars = 50 μm. Cell fusion index of osteoclast-like cultures (**B**) in contact with α-MEM and extracts from selected scaffolds: Sc (1:1, 1:2) and Sc Aln (1:1, 1:2) and in exposed to Aln solutions of 5, 2.5 µg/ml. Mean ± S.D. *P < 0.05, **P < 0.01.

Multinucleated cells positively stained for TRAP were formed after 15 days in αMEM (Fig. 7A). They were round and well-spread with fusion index of 48 ± 4% (Fig. 7B). Similar results were obtained for the extracts from reference scaffolds (Fig. 7A, Sc, first column, Fig. 7B, Sc 1:1, Sc 1:2). The addition of increasing amount of Aln to the medium resulted in the reduction of cell fusion as well as changes in cell morphology; there were clearly more spindle-shaped cells, which were not as closely packed as in the case of reference scaffolds (Fig. 7, Aln, last column). For 0.5 and 1 µg/ml Aln concentration cell morphology was similar to that of the reference samples, but for 2.5 and 5 µg/ml Aln cell fusion was inhibited and fusion index was reduced to 7 ± 3% (Fig. 7B). In the case of the extracts from scaffolds with MPs containing Aln, it was observed that the morphology of the cells resembled that of cells cultured in pure drug solutions: the higher dilution the less spindle-like cells and more giant, multinucleated cells were formed (Fig. 7A, Sc Aln, second column). Sc Aln samples at dilutions 1:1 and 1:2 as well as samples containing 5 and 2.5 µg/ml Aln looked similar: there were many spindle-like cells, while big (over 100 µm in diameter), well-spread multinucleated cells were rare in comparison with the reference Sc samples. For higher dilutions and lower concentrations of Aln, there was no significant difference between them and reference groups. Those observations correspond with TRAP 5b activity measurements.

## Discussion

The aim of this study was to improve the bioactivity of TiO_2_ scaffolds in order to enhance regeneration of bone tissue by decreasing osteoclast response and without hampering osteoblast viability. To this end, the scaffolds containing Aln-loaded degradable MPs were attached to the scaffolds’ pore walls with a thin layer of collagen. The modified scaffolds were evaluated to characterize their microstructure, drug release kinetics, cytocompatibility with osteoblast-like cells and their influence on osteoclastogenesis.

MPs were produced from biodegradable PLGA, considered safe for controlled drug delivery formulations [50]. PLGA 85:15 was chosen, because its degradation rate is slower than polymers with lower ratio of L-lactide to glycolide, e.g. 75:15 or 50:50. Moreover, relatively high molecular weight of the polymer slowed down hydrolytic degradation of the polymer matrix, which is responsible for further sustained release phase as shown earlier [44]. PLGA MPs were of 12.5 μm of median diameter and were characterized by good encapsulation and drug loading efficiencies: 71 ± 6% and 7.7 ± 0.8%, respectively. The results correspond to other formulations of polymeric MPs with Aln reported in literature [42, 43].

As shown from our previous studies the TiO_2_ scaffolds are characterized by an open porosity of about 90% and size of pores about 430 µm and were made of 100% rutile. Microstructure and mechanical properties of the scaffolds mimicked those of trabecular bone as shown earlier [51, 52]. Low concentration of collagen (40 μg/ml) proved to be sufficient to bind the MPs to the surface of the scaffolds. Collagen is known to support osteoblast-like cells, as shown by the authors’ previous work on similar collagen coatings on PLGA scaffolds [53]. Collagen provides cell adhesion polypeptide domains, e.g. GxOGER motifs which improve cell adhesion, proliferation and osteogenic differentiation [54, 55]. In this study recombinant human collagen was used, which has several advantages over bovine or porcine collagen, such as the same sequence of amino acids, no risk of pathogen transmission and better biocompatibility than collagens derived from animal tissues [56]. However, *in vivo* degradation of collagen [57] and possible detachment of the MPs from the scaffold surface must be taken into consideration. Nevertheless, even if collagen degrades upon implantation of the scaffold, the MPs delivered with the scaffold should remain within bone defect. Further *in vivo* studies are planned to evaluate the behaviour and fate of the developed implant in living organisms.

The amount of MPs attached to each scaffold was adjusted taking into account drug loading efficiency value in such a way that total encapsulated amount of Aln per scaffold was 200 µg. The applied dose was significantly lower than the recommended dose of Aln for oral (70 mg every week) or intravenous (4 mg during 15-min infusion every 3 weeks) administration [58, 59]. Aln release study showed burst of the drug from the system (48 ± 4 µg from each scaffold during 24 h, which provided 24 µg/ml concentration of Aln in non-diluted extract). The initial burst release may be associated with residual Aln adsorbed on the surface of MPs [44]. However, from Day 3 to Day 18 a zero order kinetics with 2.5 ± 0.5 µg/ml of Aln per day was achieved. Subsequently the released dose decreased up to 44 days. During the first 2 months 90% of encapsulated drug was released.

*In vitro* studies proved that Aln added to culture medium at concentrations of 10–100 µg/ml was cytotoxic for MG-63 cells: only single cells stained green were visible on the surface. Interestingly, we hardly observed red, dead cells, because they were poorly attached to the substrate and as a result they were washed out during staining procedure. Correia et al. investigated cytotoxicity of Aln different concentrations in contact with human periodontal ligament fibroblasts. They found that a concentration of 10^−5^ M (which corresponds to ca. 2.7 µg/ml) resulted in cell death after Day 6 of culture [60]. Another study performed by Garcia-Moreno et al. showed that Aln concentrations above 10^−3^ M (270 µg/ml) significantly decreased viability of the human osteoblasts [41]. In our study, non-diluted extracts and extracts diluted by factor of 2 and 4 showed some degree of toxicity, especially at shorter culture time (1 and 3 days). Taking into account release studies in PBS we can estimate that during 24 h the system provided 24 µg/ml of Aln (see Fig. 3). Thus, this concentration was too high to assure cytocompatibility with osteoblast-like cells. Nevertheless, dilutions by a factors of 4 and 8, especially for longer cell culture time periods (7 days), i.e. providing 3–6 µg/ml of Aln were found to be safe for MG-63 cells. The fact that the experiment was carried out in static conditions must be taken into consideration, because *in vivo* conditions can diminish this unwanted effect.

The results of the monocytes differentiation towards osteoclasts showed that for short culture time (8 days) the addition of Aln to the medium (in concentrations from 0.5 to 5 µg/ml) or culture of the cells in the extracts from the scaffolds was found to reduce TRAP 5b activity. This indicates that Aln released from the scaffolds inhibited osteoclastogenesis process. This effect lasted for longer culture time (15 days) and was visible especially for Aln concentrations of 2.5 and 5 µg/ml and for extracts from the scaffolds (non-diluted and diluted by factor 2). This corresponds to the results obtained by other groups [61–63]. Cenni *et al.* [63] developed PLGA-based nanoparticles loaded with Aln (0.64 µg/ml—low dose, or 6.4 µg/ml—high dose). It was found out that low dose of the nanoparticles was not cytotoxic for cells; however, it was insufficient to effectively prevent osteoclast formation.

The results of TRAP 5b activity indicate the inhibitory effect of pure Aln and the developed systems based on TiO_2_ scaffolds decorated with Aln-loaded microcarriers on the differentiation and activity of osteoclasts. The *in vitro* results performed on diluted extracts show that it is feasible to control the effective dose of the drug provided by the system by attaching to the scaffolds required amount of PLGA MPs loaded with defined amount of Aln. Thus, the developed system was found on one hand, to inhibit osteoclast differentiation and on the other hand, not to affect osteoblasts viability, what may be beneficial in the treatment critical-size bone tissue defects. However, more advanced *in vitro* and *in vivo* experiments are required to confirm the usefulness of our approach. The studies will be focused on further optimization of our system to provide adequate release kinetics of Aln and to achieve the desired enhancement in healing of critical-size defects in craniofacial region.

Having previously developed the system based on TiO_2_ scaffolds with immobilized PLGA-based MPs loaded with antibacterial agents [14, 17], it would be of great value to combine such MPs with described Aln-loaded MPs to further extend the functionally of TiO_2_ scaffolds. Such scaffolds decorated with PLGA microcarriers loaded with both antibiotics (i.e. gentamicin or vancomycin) and sodium Aln would provide structural support for osteoblasts, prevent bacterial infections and inhibit osteoclast formation.

## Conclusions

In this study, it was proved that it is possible to successfully immobilize Aln-loaded MPs on the TiO_2_ scaffolds pore walls with the use of human recombinant collagen of low concentration (40 μg/ml). The system assured controlled, prolonged release of Aln. *In vitro* tests showed that Aln concentrations of 10–100 µg/ml were found toxic for osteoblasts, but those of 5 and 2.5 µg/ml did not hamper osteoblasts viability, but reduced differentiation of monocytes derived from PBMCs towards osteoclasts. Thus the feasibility to design the system providing defined doses of Aln inhibiting osteoclastogenesis, but not affecting osteoblast functions was demonstrated. However, to confirm medical usefulness of our system more advanced *in vitro* and *in vivo* studies should be performed.
